# PLNet: Persistent Laplacian neural network for protein–protein binding free energy prediction

**DOI:** 10.1002/pro.70377

**Published:** 2025-11-20

**Authors:** Xingjian Xu, Chunmei Wang, Guo‐Wei Wei, Jiahui Chen

**Affiliations:** ^1^ Department of Mathematics University of Florida Gainesville Florida USA; ^2^ Department of Mathematics Michigan State University East Lansing Michigan USA; ^3^ Department of Electrical and Computer Engineering Michigan State University East Lansing Michigan USA; ^4^ Department of Biochemistry and Molecular Biology Michigan State University East Lansing Michigan USA; ^5^ Department of Mathematical Sciences University of Arkansas Fayetteville Arkansas USA

**Keywords:** binding free energy, deep learning, persistent homology, protein‐protein interaction

## Abstract

Recent advances in topology‐based modeling have greatly improved molecular prediction tasks, particularly in protein–ligand binding affinity. However, when the focus shifts to predicting protein–protein interactions (PPIs) binding free energy, the question becomes significantly more challenging due to the ineffective use of topological features and the lack of reliable datasets. In this work, we propose a persistent‐Laplacian machine learning framework centered on the Persistent‐Laplacian Neural Network (PLNet), which encodes each protein chain at the binding interface using both persistent Laplacian–based features and protein language model embeddings. It can achieve a promising Pearson correlation of 0.80 under leave‐out‐protein‐out cross‐validation on our newly assembled benchmark dataset, P2P, which includes 6886 protein complexes drawn from existing sources. For comparison, we also implement a gradient‐boosting decision tree model under the same settings. This baseline method highlights the advantage of PLNet in capturing complex topology‐aware descriptors in PPI prediction.

## 1 INTRODUCTION

Protein‐protein interactions (PPIs) underlie diverse biological processes, including cell metabolism, signal transduction, muscle contraction, and immune responses (Blazer & Neubig, 2009; Lu et al., 2020; Mabonga & Kappo, 2019). In therapeutic contexts, high‐affinity PPIs are central to antibody–antigen recognition and the efficacy of antibody‐based drugs (Ausserwöger et al., 2022; Ben‐Kasus et al., 2007; Bostrom et al., 2009; Kubiak‐Ossowska et al., 2020; Petta et al., 2016). Precision medicine leverages molecular insights to tailor interventions and reduce adverse effects, often by targeting PPIs implicated in disease mechanisms (Collins & Varmus, 2015; Hood et al., 2012). Consequently, accurate characterization of PPI binding affinity is critical for designing biologics and other therapeutics. However, prediction remains challenging due to conformational dynamics, post‐translational modifications, heterogeneous environments, and model limitations that collectively complicate faithful representation of protein interactions (Dill & MacCallum, 2012; Feig et al., 2004; Jensen, 2004). Additional difficulties arise from the structural diversity of proteins and variability in experimental conditions (Almo et al., 2013; Anfinsen, 1973).

Three‐dimensional (3D) structures provide essential topological and physical information about PPIs, including binding sites, conformational changes, and the spatial arrangement of key residues (Geng et al., 2019; Goodsell & Olson, 2000). The RCSB Protein Data Bank (PDB) contains a large and growing collection of protein–protein complex structures (Berman et al., 2000; Rose et al., 2015), enabling analysis of interaction mechanisms at atomic resolution. Complementary thermodynamic measurements—such as binding affinity, enthalpy, and entropy—quantify interaction strength and stability. Integrating structural and thermodynamic data supports mechanistic understanding and the design of inhibitors, activators, and therapeutic agents. Experimentally, X‐ray crystallography, NMR spectroscopy, and cryo‐EM remain primary methods for determining complex structures (Cheng, 2018; Wüthrich, 2003).

The expansion of structural and computational resources has supported the construction of diverse datasets for PPI modeling, broadly categorized into binding free energies (ΔG) and mutation‐induced changes (ΔΔG). The PDBbind database, for example, reports binding affinities for multiple classes of complexes and includes a PPI subset (2852 complexes in the 2020 release) (Liu et al., 2015, 2017; Su et al., 2018). While PDBbind has been widely used for protein–ligand studies (Ballester & Mitchell, 2010; Cang et al., 2018; Nguyen & Wei, 2019; Volkov et al., 2022), its PPI subset has been less utilized due to incomplete binding‐partner annotations and limited curation, underscoring the need for expanded, standardized collections. Datasets focused on ΔΔG provide paired measurements for wild‐type and mutant proteins and are valuable for understanding mutation effects and guiding protein engineering (Jankauskaitė et al., 2019; Moal & Fernández‐Recio, 2012; Sirin et al., 2016). SKEMPI v2, for instance, catalogs 7085 mutations in structurally characterized PPIs with associated affinity changes (Jankauskaitė et al., 2019; Moal & Fernández‐Recio, 2012). These resources are essential for benchmarking predictive models and enabling systematic assessment of mutations, including those in viral proteins such as SARS‐CoV‐2 spike.

Computational methods have become essential tools for calculating the binding affinity of PPIs, drawing upon the principles of molecular dynamics and quantum mechanics. Rigorous approaches such as free energy perturbation (FEP) and thermodynamic integration have been utilized to compute PPI interaction energies; however, these methods are computationally expensive and often face convergence challenges due to the necessity of simulating numerous nonphysical intermediate states (Bash et al., 1987; Jorgensen & Thomas, 2008; Kita et al., 1994; Kollman, 1993; Zacharias et al., 1994). To address these limitations, alternative techniques like linear interaction energy and molecular mechanics methods, notably the MM/GBSA approach, have gained popularity for their balance between accuracy and computational efficiency (Cristian Obiol‐Pardo et al., 2008; Huo et al., 2002; Massova & Kollman, 1999; Ylilauri & Pentikaïnen, 2013; Zuo et al., 2012). Additionally, empirical methods—including force‐field potentials, statistical potentials, and scoring functions used in protein docking—have evolved to offer faster predictions and have paved the way for integrating machine learning techniques to enhance accuracy without significantly increasing computational costs (Abbasi et al., 2018, 2020; Bryant et al., 2022; Chéron et al., 2017; Dvs & Ron, 2010; Huang et al., 2020; Liu et al., 2004; Vangone & Bonvin, 2015; Wang, Su, & Wu, 2021; Yugandhar & Gromiha, 2014). For instance, tools like PRODIGY leverage structural and functional features, such as networks of interfacial contacts, to predict binding affinities using regression models (Vangone & Bonvin, 2015). CP_PIE employs scoring functions and includes benchmark datasets for evaluation, enhancing its utility in predicting PPI affinities (Dvs & Ron, 2010). Techniques like DFIRE utilize statistical potentials derived from docking studies to assess protein–peptide and protein–protein complexes (Liu et al., 2004). Machine learning approaches have also been integrated, with methods like PPI‐Affinity using support vector machines and mmCSM‐PPI applying Monte Carlo simulations to reconstruct binding affinities by decomposing contributions from interfacial and non‐interfacial residues (Wang, Su, & Wu, 2021). Sequence‐based methods, such as ISLAND (Abbasi et al., 2020) and PPA‐pred (Yugandhar & Gromiha, 2014), predict binding affinities using amino acid sequence information, expanding the toolkit available for PPI analysis. A minimal yet crucial discussion of binding site identification methods underscores the importance of accurately determining interaction interfaces, which is fundamental for improving the reliability of binding affinity predictions.

Persistent homology, a cutting‐edge area within algebraic topology, bridges geometry and topology to unveil the underlying structures of complex systems (Edelsbrunner & Harer, 2010; Zomorodian & Carlsson, 2004). Element‐specific persistent homology addresses limitations by preserving essential biological details during topological abstraction, thereby facilitating a deeper understanding of protein–protein interactions upon mutations (Wang, Cang, & Wei, 2020). This approach offers innovative ways to characterize and comprehend the complex topological features of protein complexes. However, persistent homology alone may not be sufficient for representing protein complex data comprehensively. This challenge in TDA was addressed by the introduction of the persistent Laplacian, or persistent spectral graph theory (Wang, Nguyen, & Wei, 2020). The persistent Laplacian manifests the full set of topological invariants and captures the shape of data through its harmonic and non‐harmonic spectra, respectively. Additional mathematical analyses (Mémoli et al., 2022) and software packages like HERMES (Wang, Zhao, et al., 2021) have been developed to support persistent Laplacian methods. This approach has been successfully applied to biological studies, including protein thermal stability (Wang, Nguyen, & Wei, 2020), protein–ligand binding (Meng & Xia, 2021), and protein–protein binding with mutation problems (Chen et al., 2022).

This study introduces PLNet, a machine learning framework for predicting PPI binding affinities using binding‐interface features extracted from PDB structures. The input representation integrates multiscale topological descriptors from persistent homology and the persistent Laplacian, together with standard physicochemical properties and protein language model embeddings. By combining these heterogeneous descriptors, PLNet leverages the complementary strengths of topological, structural, and sequence‐based information. To rigorously assess performance, we evaluate PLNet on a newly constructed benchmark dataset, P2P, which encompasses nearly all perspectives covered by existing open PDB‐based datasets. On this benchmark, PLNet achieves promising results and consistently outperforms existing methods on several important subsets. Additionally, to complement PLNet, we also design the Persistent‐Laplacian Decision Tree (PLD‐Tree), a traditional gradient‐boosting decision tree (GBDT) model built on the same input features and interface setup. Though PLD‐Tree provides a strong and interpretable baseline, PLNet's flexible neural architecture allows it to more effectively approximate binding free energies and achieves better predictive performance than PLD‐Tree. Beyond current results, PLNet offers greater extensibility, enabling future integration with advanced machine learning architectures to further improve predictive performance. Overall, the neural design of PLNet achieves the best observed accuracy in our experiments while maintaining robust generalization. Our results demonstrate that integrating persistent‐topological descriptors with modern learning models offers a principled and effective framework for predicting PPI affinity.

## 2 MACHINE LEARNING FRAMEWORK FOR PPI AFFINITY PREDICTION

**FIGURE 1:**
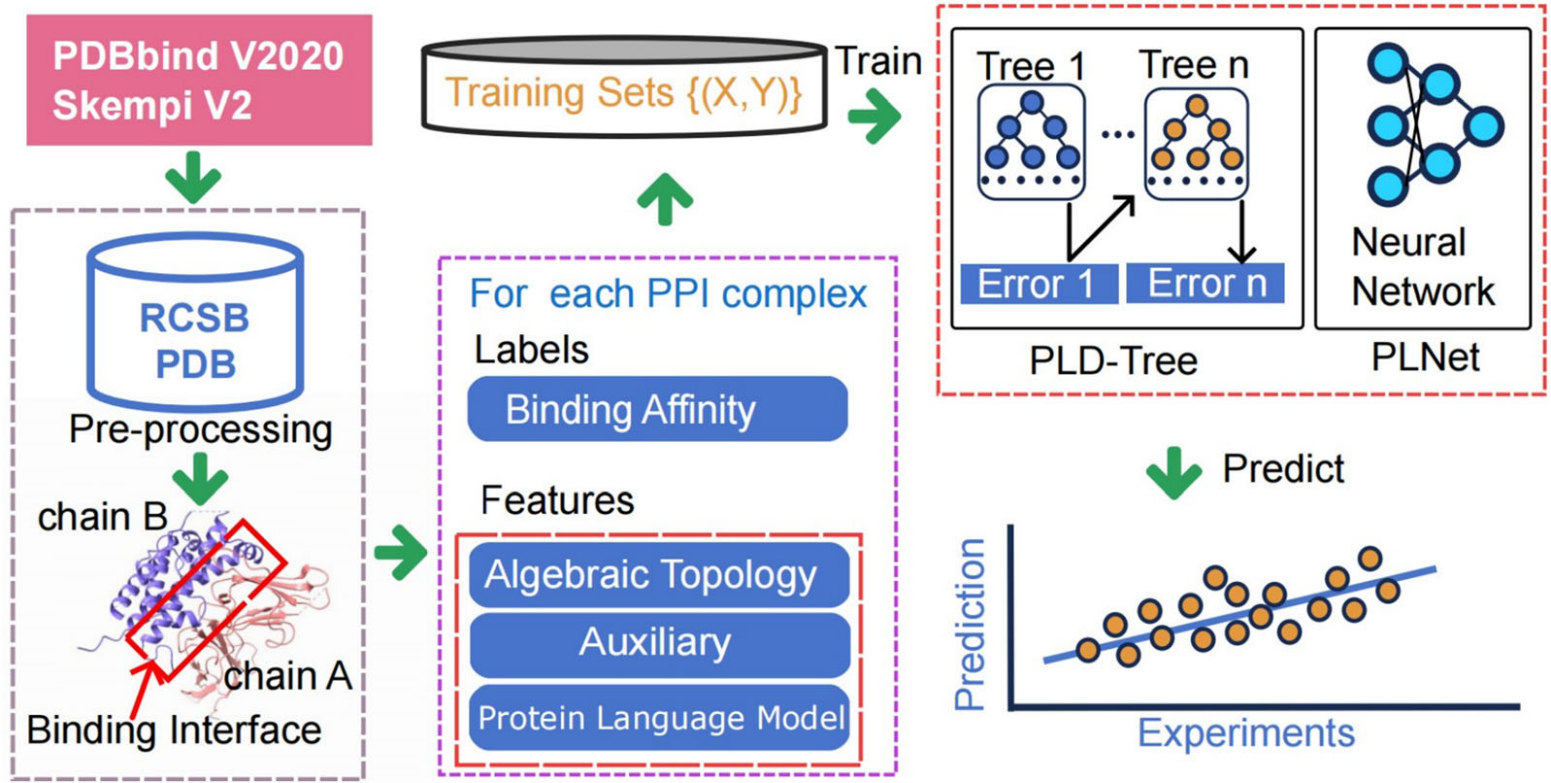
Structure of the PLNet model. Protein structures from the PDBbind V2020 and SKEMPI v2 datasets undergo preprocessing to identify binding interfaces (partners A and B). From each interface, three sets of features are extracted: Algebraic topology, auxiliary descriptors (e.g., pKa), and embeddings from protein language models. These feature sets, alongside experimental binding affinities as labels, train an ensemble of gradient‐boosting decision trees. The resulting PLNet predictor then estimates protein–protein binding affinities for new complexes.

This section outlines the proposed machine learning framework. The workflow in Figure 1 consists of topology‐based feature extraction, model construction, and prediction using PDBbind V2020 and SKEMPI v2. Here, algebraic topology provides invariants from persistent homology ($H_{0}$, $H_{1}$, $H_{2}$) and the persistent Laplacian, while auxiliary features include physicochemical descriptors and protein language model means the embeddings from evolutionary scale modeling (ESM) (Rives et al., 2019). Furthermore, Figure 1 illustrates how pairwise interactions between atoms are characterized using the zeroth homology group ($H_{0}$) for clusters and the first homology group ($H_{1}$) for loop‐like structures derived from Euclidean distance‐based filtration.

### 2.1 Data sources, auditing, and preprocessing: P2P

We assembled an integrated benchmark dataset P2P from PDBbind V2020 and SKEMPI v2 for training and evaluation. The PDBbind V2020 dataset originally contained 2852 protein–protein complexes with experimentally measured affinities. Since binding‐partner annotations are incomplete and some entries contained noisy or missing data, we manually audited each complex against the RCSB PDB to identify the interacting partners. For consistency, we retained only entries with affinities reported as $K_{d}$, $K_{i}$, or $\Delta G_{\text{bind}}$. Complexes with incomplete physical data or imprecise affinity values (e.g., reported as ranges) were excluded or, where appropriate, approximated with boundary estimates. After curation, 2368 complexes remained. Each complex was preprocessed with Profix (Xiang & Honig, 2001) to restore missing residues. We then identified the binding interface and labeled the partners as “A” and “B,” which we use consistently throughout feature construction. In addition, we calculated the pairwise sequence identity for all chains across the two partners of each complex using global Needleman–Wunsch alignments. The identity was defined as the number of identical residues divided by the alignment length; in the curated PDBbind V2020 subset used here, all pairwise chain identities satisfy ≤25%. SKEMPI v2 dataset aggregates mutation‐induced binding free energy changes (ΔΔG) from AB‐Bind, PROXiMATE, and dbMPIKT, totaling 7085 measurements across 345 PPIs with resolved complex structures. Applying a similar curation protocol, excluding ambiguous mutants and averaging repeated measurements, we obtained 343 wild‐type systems and 4175 single mutants. SKEMPI v2 provides explicit partner assignments, so no additional subdivision into A/B was required. Mutant structures were generated by introducing the specified single‐residue substitution into the corresponding wild‐type PDB structure.

Combining the curated PDBbind V2020 (2368 complexes) with SKEMPI v2 (343 wild‐type +4175 mutants) yields the P2P benchmark comprising 6886 protein complexes. For downstream analyses, we also define two subsets: P2P wt, consisting of PDBbind V2020 and SKEMPI v2 wild‐type complexes, and P2P mt, consisting of SKEMPI v2 mutant complexes together with PDBbind V2020. Distributional summaries of P2P are provided in Figure S1. Additionally, we compute pairwise sequence similarities between PDB entries and their corresponding mutants; each mutant inherits the split assignment of its parent PDB entry, and all pairs satisfy the ≤25% sequence‐identity constraint, maintaining dataset efficiency and reducing information leakage.

#### 2.1.1 Representation of binding interfaces

After curation, each PPI complex is treated as two interacting sets, A and B, corresponding to the designated partners. We select all atoms within a cutoff distance r of the interface to capture the region of intermolecular contact. Within these atoms, we consider two granularities:

1. *Element‐specific sets*: atoms grouped by elemental type.
2. *Residue‐specific sets*: atoms grouped by their amino acid residue.

#### 2.1.2 Modeling noncovalent interactions

To systematically analyze and quantify the non‐covalent interactions at the binding interface, we employed a modified distance function $D_{\mathrm{mod}}\left(a_{i},a_{j}\right)$:

(1) $$D_{\mathrm{mod}}\left(a_{i},a_{j}\right)=\left\{\begin{array}{ll} \infty , & \text{if}\;a_{i},a_{j}\in A\;\text{or}\;a_{i},a_{j}\in B\\ ∥r_{i}-r_{j}∥, & \text{if}\;a_{i}\in A\;\text{and}\;a_{j}\in B \end{array}\right.$$

where $r_{i}$ and $r_{j}$ are the position vectors of atoms $a_{i}$ and $a_{j}$, respectively, and $\left\|r_{i}-r_{j}\right\|$ is the Euclidean distance between them, which we denote as $\Phi_{\mathrm{ij}}$ for convenience. In this framework, “Partner A” and “Partner B” represent the primary components of the PPI complex. When multiple chains exist within a partner, we adopt the following conventions: (i) if only one chain participates in binding, that chain is used for its partner; (ii) nonbinding chains are ignored; and (iii) if multiple interfaces exist, we retain the primary (most inclusive) interface. By applying the modified distance function $D_{\mathrm{mod}}$ to these well‐defined sets of atoms (either element‐specific or residue‐specific), we can systematically capture the key non‐covalent interactions that govern protein–protein binding. This representation serves as a crucial step for downstream modeling tasks, including feature extraction and topological analyses aimed at predicting binding affinities.

### 2.2 Topological embedding features

Topological embedding features leverage the geometric and structural complexities of protein–protein interfaces by examining point clouds derived from atomic coordinates or amino acid positions. Through this lens, a wide array of topological invariants, including $H_{0}$ (connected components), $H_{1}$ (loops or cycles), and $H_{2}$ (voids or cavities), can be extracted. Persistent homology provides a powerful mechanism for capturing these invariants across varying scales, while the persistent Laplacian further refines the analysis by integrating geometric considerations through targeted filtration processes. This combined framework yields a rich topological characterization of binding interfaces, offering deeper insights into the underlying architecture of PPIs.

Using a Rips complex constructed from the distance matrix of these complexes, we derive persistence diagrams that highlight critical topological features—clusters, holes, and other structural motifs—across multiple scales. To hone in on the most meaningful invariants, we apply a cutoff and filter out barcodes with lifespans, ensuring that only the most significant topological patterns are retained. Subsequently, barcodes falling within specified death ranges are cataloged and binned, enhancing our capacity to elucidate the topological landscape of the binding interface. To further dissect these topological features, we examine subsets of the distance matrix at progressively tighter distance thresholds (Bins), generating Laplacian matrices whose eigenvalues encode geometric and topological information. For each pair of elements in a given element list, we compute statistical descriptors of these eigenvalues, including their sum, minimum, maximum, mean, standard deviation, variance, sum of squares, and the count of significant eigenvalues. Such a statistical treatment of eigenvalues provides a multifaceted characterization of the complexes' structural and topological signatures.

This comprehensive methodological approach, outlined in Equation (1), enables the systematic examination of persistent homology and topological features at the atomic level. Expanding the same principles to the amino acid level supplies yet another layer of detail, capturing subtle differences in topological features arising from residue‐level organization. Ultimately, this topological embedding framework allows us to identify and quantify robust patterns that define the binding interfaces of PPIs, paving the way for more accurate predictions of binding free energies and an improved understanding of protein function.

### 2.3 Protein language models

Recent advancements in protein property modeling have emerged from the use of protein language models trained on extensive protein sequence datasets. Models such as ESM (Hsu et al., 2022; Meier et al., 2021; Rives et al., 2019; Zeming Lin et al., 2022) and ProtTrans (Elnaggar et al., 2021) have exhibited remarkable capabilities, which can be further enhanced by hybrid fine‐tuning strategies that incorporate both local and global evolutionary signals. In particular, the ESM model can be fine‐tuned using downstream task data or local multiple sequence alignments, yielding increasingly accurate predictions. In this study, we utilized the ESM‐2 (t33 650M UR50D) transformer model, originally trained on a dataset of 250 million protein sequences using a masked sequence prediction objective. With a 34‐layer architecture and approximately 650 million parameters, ESM‐1b generates rich sequence embeddings. For sequences that exceed the model's input length, we partitioned them into subsequences, ensuring that accurate embeddings could still be derived. This approach produced a 3840‐dimensional feature vector for each PPI complex, substantially improving the performance and predictive power of our model.

### 2.4 Auxiliary features

In addition to sequence‐derived features, we also integrated a suite of biochemical and biophysical descriptors to capture the molecular interactions governing PPIs. These features included solvent‐accessible surface area (ASA), which quantifies the portion of the protein's surface in contact with the solvent, and buried surface area, which identifies regions concealed upon complex formation. Assigning partial charges to interacting residues using methods like PDB2PQR (Dolinsky et al., 2004) enabled us to compute Coulombic interaction energies via Coulomb's law. The Lennard‐Jones potential further refined our representation by accounting for van der Waals interactions, balancing attractive and repulsive atomic forces. Additionally, we solved the Poisson‐Boltzmann equation to incorporate electrostatic effects, reflecting a physiological ionic environment and fixed protein charges using MIBPB (Chen et al., 2011). This calculation yielded electrostatic potential maps and electrostatic free energies of binding, which served as another valuable set of features. In total, our model extracted 74 such biophysical features for Partner A and Partner B, respectively. By integrating these comprehensive physical descriptors with sequence‐derived embeddings, we constructed a more robust, informative representation of PPIs, ultimately leading to deeper insights and improved predictive accuracy. Details of auxiliary feature generation are presented in Section S1.1 in Appendix S1.

### 2.5 Machine learning platform

The last part of this session focuses on the machine learning platform. Our predictive modeling approach utilizes multilayer perceptrons (MLPs), which consistently provided higher predictive accuracy and more stable generalization than tree‐based baselines, including GBDT. The superior performance of MLPs, together with their ability to capture nonlinear interactions among features, made them our method of choice for $K_{d}$ prediction of PPI complexes. We implemented feed‐forward MLPs in PyTorch (Paszke et al., 2019) with ReLU activations and dropout for regularization. Training was performed using the Adam optimizer with early stopping on a held‐out validation set to prevent overfitting. Model depth, width, and hyperparameters were tuned via grid search. The optimal architecture was a six‐layer MLP with 400 neurons per layer and ReLU activations. Additional details on PLD‐Tree are provided in Tables S2–S6 and Figures S1–S3.

## 3 RESULTS AND DISCUSSION

In the following subsection, we evaluate our models using Pearson's correlation coefficient ($R_{p}$), mean absolute error (MAE) and root mean squared error (RMSE). We compute $R_{p}$ not only on the training data (10‐fold cross‐validation) but also on an independent development set. By combining both correlation and error metrics, we ensure a comprehensive understanding of the model's predictive capabilities. Pearson's correlation coefficient $R_{p}$ is defined as:

(2) $$R_{p}=\frac{\sum_{i=1}^{n}\left(y_{i}-\bar{y}\right)\left(y_{i}^{\text{pred}}-{\bar{y}}^{\text{pred}}\right)}{\sqrt{\sum_{i=1}^{n}{\left(y_{i}-\bar{y}\right)}^{2}\sum_{i=1}^{n}{\left(y_{i}^{\text{pred}}-{\bar{y}}^{\text{pred}}\right)}^{2}}}$$

Here, $y_{i}$ and $\bar{y}$ represent the actual affinity values and their mean, respectively, while $y_{i}^{\text{pred}}$ and ${\bar{y}}^{\text{pred}}$ are the predicted values and their mean. Using both correlation‐ and error‐based metrics, we gain a thorough and reliable perspective on model performance, ultimately ensuring meaningful predictions of binding affinities.

### 3.1 Performance of the protein–protein benchmark model

In this section, we present additional experiments that illustrate the model's versatility and further validate its performance. We conducted three benchmark tests utilizing three distinct benchmark datasets, designated S79 (Vangone & Bonvin, 2015), S90 (Romero‐Molina et al., 2022; Vangone & Bonvin, 2015), and S177 (Romero‐Molina et al., 2022). The first benchmark set, S79, is from the web server PRODIGY (Vangone & Bonvin, 2015). The second benchmark set, S90, is a carefully curated subset of the refined PDBbind V2020 dataset (Romero‐Molina et al., 2022). The final benchmark set, S177, encompasses 26 wild‐type PPI complexes alongside 151 mutant complexes (Romero‐Molina et al., 2022). By evaluating PLNet and PLD‐Tree across these three benchmark datasets, we ensure a comprehensive validation of its predictive performance in diverse and challenging contexts. This multi‐dataset approach allows us to compare our framework against existing state‐of‐the‐art methods effectively, demonstrating its robustness and versatility in handling both wild‐type and mutant PPI complexes.

The ICs/NIS‐based predictor is implemented in the web server PRODIGY (Vangone & Bonvin, 2015), which estimates binding affinity (BA) using two structural descriptors: the network of inter‐residue contacts (ICs) and the noninteracting surface (NIS), which is a classical method. On a benchmark set of 79 protein–protein complexes, PRODIGY achieved an $R_{p}$ of 0.74 and an MAE of 1.4 kcal/mol, outperforming other methods. To evaluate the performance of our PLNet model, we employed the same benchmark set (Table 1 test set S79) and compared it against PRODIGY, and three other top‐ranked tools available at the time, and the ISLAND method. The traditional PLD‐Tree attained a Pearson correlation coefficient of $R_{p}=0.71$ and an MAE of 1.4 kcal/mol on the S79 test set, while our ensemble PLNet model attained the highest correlation $R_{p}=0.77$ with a slightly lower MAE of 1.3 kcal/mol, indicating both stronger linear association and improved prediction accuracy. This result highlights the strength of PLNet in capturing complex patterns in binding affinity prediction, achieving state‐of‐the‐art performance on challenging benchmark sets. This demonstrates the effectiveness of our approach, particularly in maintaining prediction precision despite the lower correlation on small test sets.

**TABLE 1:** The performance for $R_{p}$ and MAE between experimental and predicted binding affinities on the S79 and on the S90.

Method	S79		S90	
	$R_{p}$	MAE (kcal/mol)	$R_{p}$	MAE (kcal/mol)
DFIRE (Liu et al., 2004)	0.60	4.6	0.10	25.4
CP—PIE (Dvs & Ron, 2010)	−0.50	8.8	−0.10	11.0
ISLAND (Abbasi et al., 2020)	0.38	2.1	0.27	2.2
PPI‐affinity (Romero‐Molina et al., 2022)	0.62	1.8	0.50	1.8
PLD‐Tree	0.71	1.4	0.67	1.4
PLNet	0.77	1.3	0.68	1.4

Furthermore, we benchmarked our machine learning framework on Test Set S90, a subset of the P2P dataset introduced in (Romero‐Molina et al., 2022), to further evaluate its predictive performance. As shown in Table 1, our PLNet model outperforms all existing methods, achieving a Pearson correlation coefficient of $R_{p}=0.68$ and a mean absolute error (MAE) of 1.4 kcal/mol. For comparison, while the PLD‐Tree model yields a slightly lower correlation ($R_{p}=0.67$), it still surpasses all previously reported baselines. In contrast, other predictors such as PRODIGY, DFIRE, and CP_PIE experienced a substantial decline in their performance on Test Set S90 compared to Test Set S79 (Table 1). For instance, PRODIGY's performance dropped to $R_{p}=0.31$ and MAE = 2.5 kcal/mol, highlighting potential overfitting issues towards the original benchmark set. This dramatic decay suggests that these models may not generalize well to more diverse or larger datasets. Conversely, PPI‐Affinity maintained relatively consistent performance across both test sets, although it still did not match the PLNet's results.

The superior performance of this machine learning framework on Test Set S90 underscores the advanced predictive capabilities of our persistent homology‐based machine learning approach for PPIs. Moreover, our model's $R_{p}$ exceeding 0.50 not only outperforms the previously best‐known result reported by PPI‐affinities (Romero‐Molina et al., 2022) but also reaffirms the effectiveness of the topology‐based method in accurately predicting binding affinities. However, the analysis of other predictors is complicated by the absence of a defined applicability domain, which limits our ability to determine whether test samples fall outside the models' effective scope or if specific structural factors adversely impact prediction quality. Nonetheless, the robust performance of this method across multiple benchmark sets highlights its potential as a reliable and generalizable tool for PPI binding affinity prediction, especially for PLNet.

**FIGURE 2:**
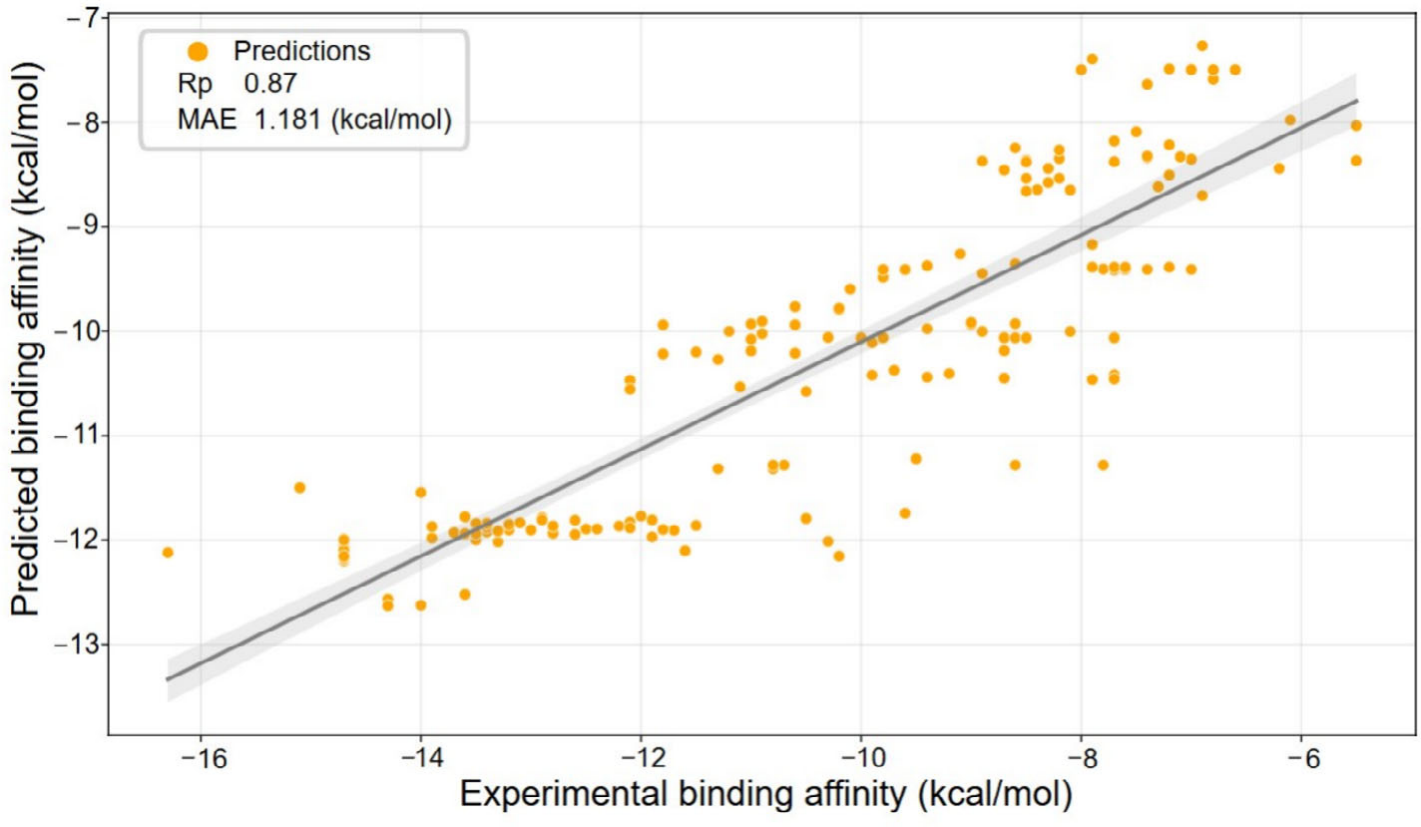
The performance of PLNet for $R_{p}$ of 0.87, MAE of 1.1 kcal/mol and scatter plot between experimental and predicted binding affinities on S177.

The third benchmark set, S177, is also derived from the PPI‐Affinity dataset (Romero‐Molina et al., 2022). Approximately 80% of the mutant complexes in this set correspond to single‐mutation structures already represented in P2P. However, S177 is more challenging, since it also includes protein–protein complexes with multiple mutations ranging from per protein sequence. This benchmark is specifically designed to evaluate how well a single model can capture generalizable features across a wide spectrum of mutation scenarios (Romero‐Molina et al., 2022), thus providing a rigorous assessment of model accuracy under increasingly complex mutation patterns. PLNet model achieves a Pearson correlation coefficient of $R_{p}=0.87$ and an MAE of 1.18 kcal/mol (Figure 2 and Table 2), outperforming both the PPI‐Affinity model ($R_{p}=0.78$, MAE = 1.40 kcal/mol) and PLD‐Tree baseline ($R_{p}=0.83$, MAE = 1.17 kcal/mol). These results demonstrate that our approach surpasses all benchmark methods presented in the referenced study. Notably, even without explicit training on data containing multi‐mutant complexes, our model delivered compelling and superior predictive performance. This further highlights the robustness and versatility of our framework in handling diverse and complex PPI scenarios.

**TABLE 2:** The result for $R_{p}$ and MAE between experimental and predicted binding affinities on S177.

Methods	$R_{p}$	MAE (kcal/mol)
PPI‐Affinity	0.78	1.4
PLD‐Tree	0.83	1.1
PLNet	0.87	1.1

### 3.2 Performance of training sets

In this section, we evaluate our models using 10‐fold cross‐validation on the PDBbind v2020 dataset, the SKEMPI wt, mt subset, and P2P, as shown in Table 3. Specifically, the leave‐one‐protein‐out strategy is applied to all SKEMPI v2 subsets and P2P to avoid cases where the same protein–protein complex appears in both the training and test sets through different mutations. When trained and evaluated solely on the PDBbind v2020 dataset, PLD‐Tree achieves the best performance among the tested models, with PLNet ranking second. However, on the SKEMPI wt and mt subsets, we observe a notable improvement in PLNet's predictive performance. On the P2P dataset, PLNet significantly outperforms PLD‐Tree, achieving an $R_{p}$ of 0.80, an MAE of 1.230 kcal/mol, and an RMSE of 1.788 kcal/mol.

**TABLE 3:** The performance for $R_{p}$ and MAE between experimental and predicted binding affinities on training sets for PDBbind V2020, SKEMPI v2, and SKEMPI v2 wild type (wt) and mutant (mt) are presented separately.

Datasets	Size	PLNet		PLD‐Tree	
		$R_{p}$	MAE (kcal/mol)	$R_{p}$	MAE (kcal/mol)
PDBbind V2020 (Liu et al., 2015)	2368	0.60	1.610	0.64	1.464
SKEMPI v2 wt (Moal & Fernández‐Recio, 2012)	343	0.71	1.543	0.68	1.533
SKEMPI v2 mt (Moal & Fernández‐Recio, 2012)	4175	0.78	1.526	0.74	1.670
SKEMPI v2 (Moal & Fernández‐Recio, 2012)	4518	0.78	1.538	0.78	1.619
P2P	6886	0.80	1.348	0.78	1.456

**FIGURE 3:**
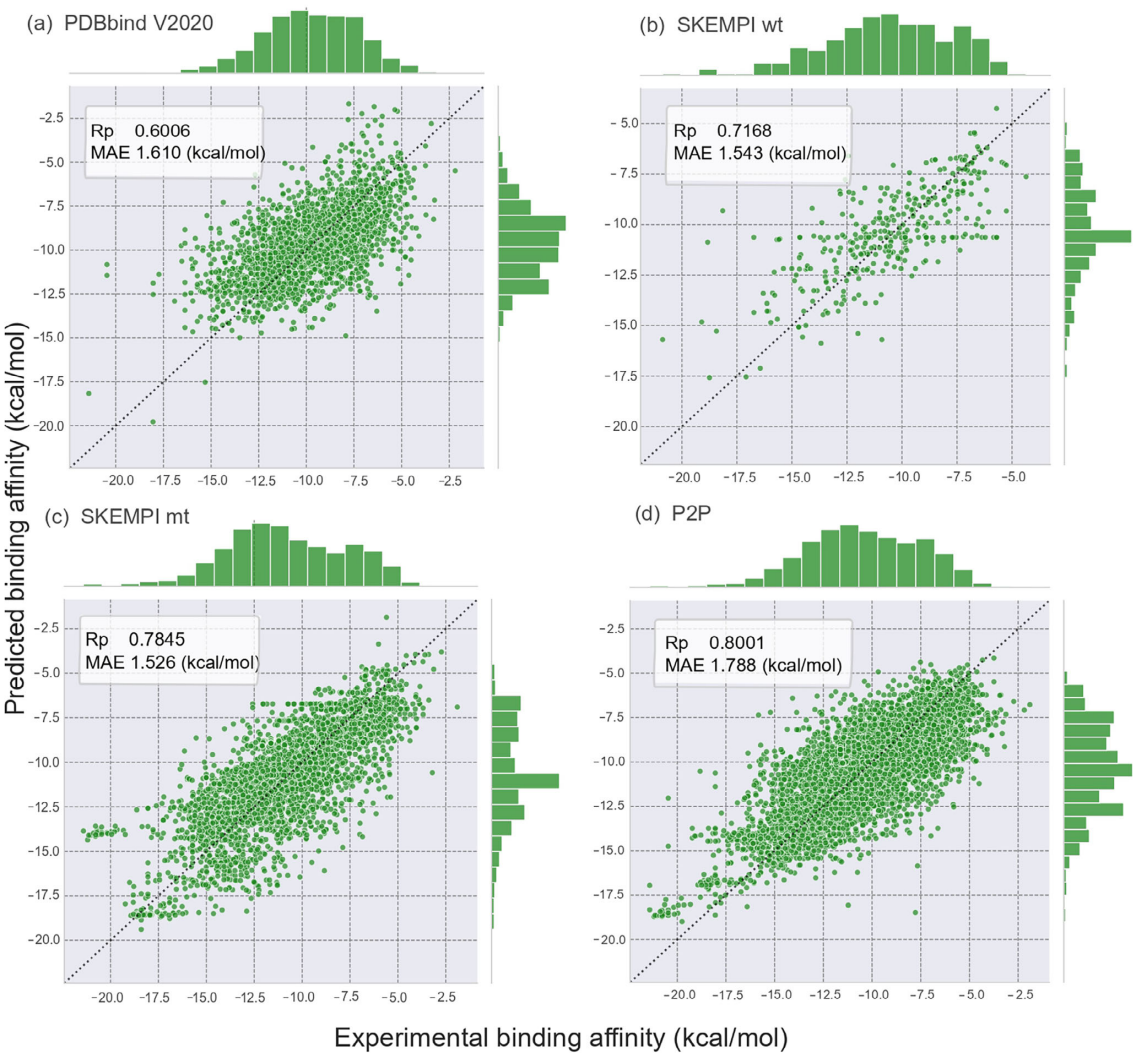
The performance of scatter plots using PLNet model on the training sets for PDBbind V2020, and SKEMPI v2.

Moreover, Figure 3 presents scatter plots for the four datasets, illustrating consistently strong results across each partition. Although the SKEMPI mt dataset exhibits the highest correlation (Figure 3c), a few outliers remain visible; by contrast, the P2P dataset shows fewer outliers and further improves cross‐validation accuracy. This substantial gain in predictive power exceeds previously reported results and underscores the effectiveness of our feature optimization strategy. To our knowledge, this is the first time such a high correlation has been achieved on the PDBbind V2020 dataset, highlighting the robust ability of PLNet to integrate diverse data sources and capture more accurately the underlying determinants of protein–protein binding affinity.

### 3.3 Performance of binding partners classification for PDBbind V2020

Unlike the SKEMPI V2 dataset, the original PDBbind V2020 dataset is more recent and does not provide explicit binding partner information. Before partitioning all PPI affinities at the binding interface into two partners—a process further complicated by symmetrical structures and multiple protein chains—we first relied on three‐dimensional structural data from the RCSB PDB. We then categorized the PPI complexes in the original PDBbind V2020 dataset into different binding classes. As summarized in Table 4, the dominant category features two binding partners (2414 complexes), many of which exhibit symmetrical structures involving DNA, RNA, or viral components.

**TABLE 4:** The size of datasets on PDBbind V2020 grouped by different numbers of binding proteins.

Binding proteins	1	2	3	4	5	>5
Dataset size	6	2414	123	190	107	12

### 3.4 Performance for antibody–antigen classification of PDBbind V2020

Developing new pharmaceutical compounds is a lengthy, costly, and resource‐intensive process. In support of drug design, it is also valuable to assess datasets consisting exclusively of antibody and antigen structures. Accordingly, we identified each chain in the PDBbind V2020 database and subset wt of the SKEMPI v2 database to extract 630 relevant complexes (hereafter denoted S630), as well as an additional 55 wild‐type records (hereafter S55). The detailed distribution of these datasets can be found in Figure S4.

We evaluated the performance of PLNet and PLD‐Tree on antibody–antigen datasets using 10‐fold cross‐validation, with results summarized in Table S1. When trained and tested solely on the S630 dataset, PLD‐Tree achieved an $R_{p}$ of 0.4925, an MAE of 1.457 kcal/mol, and an RMSE of 1.910 kcal/mol. After augmenting S630 with the S55 dataset to form S685, performance improved further, reaching an $R_{p}$ of 0.5259, an MAE of 1.395 kcal/mol, and an RMSE of 1.856 kcal/mol. In contrast, PLNet underperformed relative to PLD‐Tree on these datasets. It achieves an $R_{p}$ of 0.449, an MAE of 1.555 kcal/mol, and an RMSE of 2.036 kcal/mol in S630. On the combined S685 set, PLNet reached an $R_{p}$ of 0.464, an MAE of 1.556 kcal/mol, and an RMSE of 2.035 kcal/mol.

Even though both models yield relatively poor results, part of the reason is that some antibody–antigen complexes in the dataset exhibit low diversity and poor generalization, making it difficult for the models to effectively learn this particular class of PPIs. Although these results demonstrate some predictive capability, they also highlight the need for further improvements in feature selection and modeling strategies to enhance PLNet's accuracy on antibody–antigen data.

### 3.5 Performance between different features

Understanding the impact of different features on PPI binding affinity is essential to simplify future feature selection and representation strategies in protein engineering. To explore this, we analyzed how closely the model's predictions reflect experimental data distributions when trained on individual feature categories. Specifically, we compared the performance of our model using PPI topological features, ESM sequence embeddings, and auxiliary physicochemical descriptors on the P2P dataset and its subsets. Table 5 summarizes these results, including performance metrics obtained through a 10‐fold cross‐validation on the PDBbind V2020, P2P wt, P2P mt, and P2P dataset for each feature subset. Here, P2P wt is PDBbind V2020 merged with the SKEMPI wt, and P2P mt is PDBbind V2020 merged with the SKEMPI mt. Additionally, Table S3 introduces these features as applied to the SKEMPI v2 dataset as well.

**TABLE 5:** Performance for $R_{p}$ and MAE by different features on the considered datasets.

10‐fold	PDBbind V2020		P2P wt		P2P mt		P2P	
	$R_{p}$	MAE	$R_{p}$	MAE	$R_{p}$	MAE	$R_{p}$	MAE
PLNet								
Auxiliary	0.5412	1.668	0.5593	1.774	0.7184	1.601	0.7356	1.549
PPI	0.4265	1.888	0.4674	1.860	0.6273	1.871	0.6121	1.858
ESM	0.5822	1.578	0.6024	1.610	0.7562	1.475	0.7501	1.465
All	0.6010	1.610	0.6028	1.610	0.7765	1.429	0.8001	1.348
PLD‐Tree								
Auxiliary	0.5893	1.555	0.6191	1.558	0.7088	1.638	0.7154	1.651
PPI	0.5617	1.610	0.5976	1.613	0.6775	1.750	0.6942	1.733
ESM	0.6093	1.506	0.6355	1.499	0.7448	1.502	0.7548	1.501
All	0.6424	1.464	0.6717	1.450	0.7647	1.467	0.7799	1.456

Our findings indicate that auxiliary features generally outperform topological features when the dataset is smaller (e.g., PDBbind V2020 alone). Additional descriptors likely provide more direct, empirically grounded information about intermolecular interactions, making them particularly valuable when training data is limited. However, as we scale up to larger datasets (e.g., P2P), the relative advantage of this type of feature diminishes. Under these conditions, topological and ESM‐based features can leverage the richer data environment more effectively. The ESM embeddings, trained on extensive protein sequence databases, provide a robust evolutionary and sequence‐level context that complements the structural information captured by topological features.

In essence, each type of characteristic contributes unique information on the underlying determinants of protein–protein binding. Auxiliary features like biophysical features offer a direct link to physicochemical properties, ESM embeddings provide a broad sequence‐level context, and topological representations encode essential structural details. By understanding how these feature categories shape model performance, we can make more informed decisions about integrating and prioritizing features in future predictive modeling efforts.

It is noteworthy that, under 10‐fold cross‐validation, datasets containing single‐mutation complexes consistently enhance the model's predictive performance. This improvement can be attributed to the increased diversity introduced by various single mutations in the wild‐type complexes, enabling the model to capture a broader range of variability and underlying patterns. In contrast, the other models discussed in the previous section primarily account for differences across distinct PDB structures rather than variations within the same PDB structure when specific testing data are selected. Furthermore, we observed that the P2P dataset significantly outperforms the PDBbind V2020 dataset alone, as evidenced by a substantially lower MAE in the model's predictions. This reduction in MAE highlights the enhanced performance achieved by incorporating single‐mutation data, underscoring the value of diverse mutation information in improving predictive accuracy.

## 4 CONCLUSION

In this work, we introduced PLNet, a novel network–based predictor specifically designed for PPIs. PLNet addresses key limitations observed in previous models on the PDBbind v2020 dataset and demonstrates strong predictive performance on the recently proposed and widely recognized dataset P2P. Compared to both existing methods and our newly developed GBDT‐based model, PLD‐Tree, PLNet not only achieves higher predictive accuracy but also exhibits robust and consistent performance across diverse evaluation scenarios.

Additionally, we examined this framework's capabilities on various test sets, further highlighting its reliability and adaptability. To facilitate broader application, we have made an open‐source version of PLNet and PLD‐Tree available on GitHub, featuring functionalities for screening protein complexes and engineering amino acid compositions at the interface to enhance binding affinity or identify critical mutants that may compromise stability. This versatility positions the machine learning framework as a valuable tool in the design of novel biologics and therapeutic compounds, underscoring its potential impact in advancing protein–protein interaction research and pharmaceutical development.

## 5 METHODS

In this section, we provide an overview of algebraic topology that is frequently employed to capture the geometry and topology of molecular structures (Hatcher, 2002; Edelsbrunner & Harer, 2010; Massey, 2019; Zomorodian & Carlsson, 2004). By applying these complexes to PPI binding interfaces, we can construct multi‐scale representations that serve as the foundation for both persistent homology and the persistent Laplacian.

### 5.1 Alpha complex

Alpha complexes were first introduced by Edelsbrunner and Mücke as a generalization of the Delaunay triangulation (Edelsbrunner & Harer, 2010; Edelsbrunner & Mücke, 1994; Massey, 2019; Zomorodian & Carlsson, 2004). They extend the concept of a convex hull to reveal finer geometric details of a point cloud, making them particularly suitable for representing molecular surfaces and interfaces. Formally, given a set of points $S\subset {\mathbb{R}}^{n}$, one first constructs the Delaunay triangulation of S. The alpha complex of S at scale parameter α is then derived by selectively removing simplices whose circumscribing spheres exceed radius α. In other words, as α varies from small to large values:

- For very small α, the alpha complexes capture only the tightest clusters of points, isolating them into small connected components.
- As α increases, more simplices remain in the alpha complexes, gradually adding bridges, faces, and volumes that reflect the evolving structure of the underlying point set.
- Eventually, for a sufficiently large α, the alpha complexes include the entire Delaunay triangulation, effectively replicating the convex hull of S.

This progression from coarse to fine representations provides a filtration—a nested sequence of complexes—on which one can compute topological invariants (e.g., connected components, loops, and voids). In the context of PPI, alpha complexes are valuable for identifying cavities, tunnels, and other structural features that may be crucial to binding. By examining how these features persist or disappear over changes in α, one can obtain insights into the stability and geometry of protein interfaces.

### 5.2 Rips complex

In topology, the Rips complex (also called the Vietoris–Rips complex) is another fundamental construction for translating distance information in a point set into a simplicial complex. Unlike alpha complexes, which rely on Delaunay triangulation, the Rips complex directly uses pairwise distances among points in a metric space. See more details in Edelsbrunner and Harer (2010), Massey (2019), and Zomorodian and Carlsson (2004).

Formally, let S⊂M be a set of points in a metric space $\left(M,d\right)$. For a chosen distance threshold ϵ>0, the Rips complex $R_{\epsilon }\left(S\right)$ is defined as follows:

- Each point in S corresponds to a vertex in the complex.
- A (*k*‐dimensional) simplex is formed by any k+1 points $\left(v_{0},v_{1},\ldots ,v_{k}\right)\subset S$ if and only if the distance $d\left(v_{i},v_{j}\right)\leq \epsilon$ for all 0≤i<j≤k.

By increasing ϵ from small to large, one obtains a filtration of Rips complexes. Initially, when ϵ is very small, each point stands alone with no edges. As ϵ grows, edges, triangles, and higher‐dimensional simplices appear, linking points into connected components and revealing loops, cavities, and other topological features. This scale‐dependent perspective is integral to persistent homology, as it tracks how topological features emerge and vanish over the course of filtration. In molecular modeling, the Rips complex offers a direct way to capture multi‐scale geometry from pairwise atomic or residue distances, making it a powerful tool for analyzing the binding interfaces of protein complexes.

### 5.3 Simplicial complex and filtration

An abstract simplicial complex is a finite collection of sets of points (i.e., atoms) $K={\left\{\sigma_{i}\right\}}_{i}$, where the elements in $\sigma_{i}$ are called vertices and $\sigma_{i}$ is called a *k*‐simplex if it has k+1 distinct vertices. If $\tau \subseteq \sigma_{i}$ for $\sigma_{i}\in K$ indicates that τ∈K, and that the non‐empty intersection of any two simplices $\sigma_{1},\sigma_{2}\in K$, is a face of both $\sigma_{1}$ and $\sigma_{2}$. These foundational definitions can be found in (Hatcher, 2002).

In practice, it is favorable to characterize point clouds or atomic positions in various spatial scales rather than in a fixed scaled simplicial complex representation. To construct a scale‐changing simplicial complex, consider a function f:K→ℝ that satisfies $f\left(\tau \right)\leq f\left(\sigma \right)$ whenever τ⊆σ. Given a real value, x,f induces a subcomplex of K by constructing a sub‐level set, $K\left(x\right)=\left\{\sigma \in K|f\left(\sigma \right)\leq x\right\}$. As K is finite, the range of f is also finite and the induced subcomplexes, when ordered, form a filtration of K,

0⊂Kx1⊂Kx2⊂⋯⊂Kxl=K

There are many constructions of K and one that is widely used for point clouds is the Rips complex. Given K as the collection of all possible simplices from a set of atomic coordinates until a fixed dimension, the filtration function is defined as $f_{\text{Rips}}\left(\sigma \right)=\max\left\{d\left(v_{i},v_{j}\right)|v_{i},v_{j}\in \sigma \right\}$ for σ∈K, where d is a predefined distance function between the vertices; for example, $D_{e}$. In practice, an upper bound of the filtration value is set to avoid an excessively large simplicial complex. Another efficient construction called the alpha complex is often used to characterize geometry, and we denote the filtration function by $f_{\alpha }:\mathrm{DT}\left(X\right)\to \mathbb{R}$, where $\mathrm{DT}\left(X\right)$ is the simplicial complex that is induced by the Delaunay triangulation of the set of atomic coordinates, X. The filtration function is defined as $f_{\alpha }\left(\sigma \right)=\max\left\{\left(1/2\right)D_{e}\left(v_{i},v_{j}\right)|v_{i},v_{j}\in \sigma \right\}$ for $\sigma \in \mathrm{DT}\left(X\right)$. Back to molecular structures, the filtration of simplicial complexes describes the topological characteristics of interaction hypergraphs under various interaction range assumptions.

### 5.4 Homology groups

In the context of singular homology, a homology group of a simplicial complex topologically represents hole‐like structures of various dimensions. Given a simplicial complex K, a *k*‐chain is a finite formal sum of *k*‐simplices in K; that is, $\sum_{i}a_{i}\sigma_{i}$. For simplicity, we choose coefficients $a_{i}$ from ${\mathbb{Z}}_{2}$. The *k*th chain group, denoted $C_{k}\left(K\right)$, comprises all *k*‐chains with addition induced by the addition of coefficients. The formal definition can be found in (Hatcher, 2002; MacLane, 2012).

A boundary operator $\partial_{k}:C_{k}\left(K\right)\to C_{k-1}\left(K\right)$ connects chain groups of different dimensions by mapping a chain to the alternating sum of its codimension‐1 faces. For simplices, this operator is defined as follows:

$$\partial_{k}\left(\left\{v_{0},\ldots ,v_{k}\right\}\right)=\sum_{i=0}^{k}{\left(-1\right)}^{i}\left[v_{0},\ldots ,{\hat{v}}_{i},\ldots ,v_{k}\right],$$

where ${\hat{v}}_{i}$ signifies that vertex $v_{i}$ is omitted. The *k*th cycle group, denoted $Z_{k}\left(K\right)$, is the kernel of $\partial_{k}$, and its elements are called *k*‐cycles. The *k*th boundary group, $B_{k}\left(K\right)$, is the image of $\partial_{k+1}$. By the property $\partial_{k}\circ \partial_{k+1}=0$, $B_{k}\left(K\right)$ is a subgroup of $Z_{k}\left(K\right)$. The *k*th homology group, $H_{k}\left(K\right)$, is defined as the quotient group $Z_{k}\left(K\right)/B_{k}\left(K\right)$. The equivalence classes in $H_{k}\left(K\right)$ correspond to *k*‐dimensional holes in *K* that cannot be deformed into each other by the boundary of a subcomplex.

Given a filtration as mentioned earlier, in addition to characterizing the homology group at each step $H_{k}\left(K\left(x_{i}\right)\right)$, it is important to track the persistence of topological features throughout the sequence. Viewing $H_{k}\left(K\left(x_{i}\right)\right)$ as vector spaces with inclusion map‐induced linear transformations gives a persistence module:

$$H_{k}\left(K\left(x_{1}\right)\right)\to H_{k}\left(K\left(x_{2}\right)\right)\to \cdots \to H_{k}\left(K\left(x_{\ell }\right)\right).$$

An interval module with respect to $\left[b,d\right)$, denoted $I_{\left[b,d\right)}$, is defined as a collection of vector spaces $\left\{V_{i}\right\}$ connected by linear maps $f_{i}:V_{i}\to V_{i+1}$. Here, $V_{i}={\mathbb{Z}}_{2}$ for $i\in \left[b,d\right)$ and $V_{i}=0$ otherwise. The map $f_{i}$ is the identity map when possible and zero otherwise.

The persistence module can be decomposed as a direct sum of interval modules ${⊕}_{\left[b,d\right)\in B}I_{\left[b,d\right)}$. Each $I_{\left[b,d\right)}$ corresponds to a homology class that appears at filtration value b (birth) and disappears at filtration value d (death). The collection of these pairs, B, records the evolution of *k*‐dimensional holes with varying filtration parameters, and thus captures the topological configuration of the input point cloud under different interaction ranges, especially when using a distance‐based filtration.

### 5.5 Persistent Laplacian

Persistent Laplacian, also known as persistent spectral graphs or the persistent combinatorial Laplacian (Mémoli et al., 2022; Wang, Nguyen, & Wei, 2020), emerged to address a key limitation of persistent homology—namely, its inability to capture homotopic shape evolution in data. The method relies on a filtration process, whereby a dataset is converted into a sequence of nested simplicial complexes of increasing complexity. At each filtration level, Betti numbers are computed to track topological changes (persistence) as the resolution of the data increases.

For a simplicial pair K⊆L, consider the subspace

$$C_{p}^{L,K}=\left\{c\in C_{p}^{L}:\partial_{p}^{L}\left(c\right)\in C_{p-1}^{K}\right\}\subseteq C_{p}^{L},$$

where $C_{p}^{L,K}$ consists of all simplices in the $C_{p}^{L}$, and also $C_{p-1}^{K}\subseteq C_{p-1}^{L}$ for boundary condition. We then define $\partial_{p}^{L,K}$ as the restriction $\partial_{p}^{L}|C_{p}^{L,K}$. The *p*‐persistent Laplacian is given by

$$\Delta_{p}^{K,L}=\partial_{p+1}^{L,K}\circ {\left(\partial_{p+1}^{L,K}\right)}^{*}+{\left(\partial_{p}^{K}\right)}^{*}\circ \partial_{p}^{K},$$

and its upper component is $\Delta_{p,\mathrm{up}}^{K,L}=\partial_{p+1}^{L,K}\circ {\left(\partial_{p+1}^{L,K}\right)}^{*}$. The following diagram illustrates the relationships among these boundary maps:

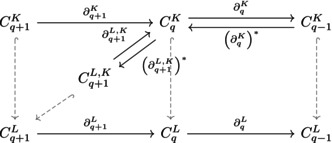

When K and L are graphs with the same vertex set, $\Delta_{0}^{K,L}$ reduces to the usual graph Laplacian $\Delta_{0}^{L}$ of the larger graph L. Further details on computation and kernel properties can be found in (Mémoli et al., 2022).

To investigate persistent spectral properties, one typically constructs a filtration $K_{0}\subset K_{1}\subset \cdots \subset K_{n}$, and then associates a Laplacian matrix (e.g., the combinatorial Laplacian ${ℒ}_{q}$) to each complex $K_{i}$. By examining how eigenvalues and eigenvectors evolve across these nested complexes, one gains insight into the topological and geometric structure of the data at multiple resolutions (Wang, Nguyen, & Wei, 2020). Crucially, the harmonic spectra of persistent Laplacians at different scales coincide with persistent Betti numbers, whereas the non‐harmonic components capture both topological and homotopic shape evolution.

## AUTHOR CONTRIBUTIONS

**Xingjian Xu:** Data curation; software; writing – original draft; visualization. **Chunmei Wang:** Writing – review and editing; funding acquisition; investigation. **Guo‐Wei Wei:** Writing – review and editing; funding acquisition; conceptualization. **Jiahui Chen:** Writing – original draft; visualization; conceptualization.

## Supporting information


**TABLE S1:** Performance of PLNet and PLD‐Tree for $R_{p}$ and MAE between experimental and predicted binding affinities on antibody‐antigen dataset S630, S55 and S685.
**TABLE S2:** Performance for PLD‐Tree of $R_{p}$ and MAE on unclassified cross‐validation by different features on the considered datasets.
**TABLE S3:** Performance of PLD‐Tree for $R_{p}$ and MAE by different features on the SKEMPI v2 dataset.
**TABLE S4:** Performance of PLD‐Tree for $R_{p}$ and MAE on P2P for hyperparameter finding (1).
**TABLE S5:** Performance of PLD‐Tree for $R_{p}$ and MAE on P2P for hyperparameter finding (2).
**TABLE S6:** Performance of PLD‐Tree for $R_{p}$ and MAE on P2P for hyperparameter finding (3).
**FIGURE S1:** Distributions of binding affinities among the three dataset PDBbind V2020, SKEMPI wt and SKEMPI mt.
**FIGURE S2:** Scatter plots of performance using the PLD‐Tree model on the training datasets for PDBbind V2020 and SKEMPI V2.
**FIGURE S3:** Scatter plots of performance using the PLD‐Tree model on the subset of P2P: P2P wt and P2P mt.
**FIGURE S4:** Distributions of binding affinities among antibody–antigen test dataset S630, S55, and S685.

## Data Availability

All data and code utilized in this study will be made publicly accessible on GitHub upon the acceptance of this paper available at: https://github.com/xxjan719/PLNet. The GitHub repository will include: Datasets: The P2P dataset consists of processed versions of the PDBbind V2020 and SKEMPI v2 datasets, along with any additional datasets used in our analysis, all of which can be found in the repository. Code: Scripts for data preprocessing, feature extraction, model training, and evaluation of the PLNet model. Documentation: Detailed instructions and guidelines to facilitate replication of our results, including dependencies and usage examples.
