# Supplementary material for: PLNet: Persistent Laplacian neural network for protein–protein binding free energy prediction
Source: Protein Sci. 2025 Nov 20;34(12):e70377. doi: 10.1002/pro.70377 (PMC12631946; doi:10.1002/pro.70377)
Supplement: Supplementary file 1 — TABLE S1: Performance of PLNet and PLD‐Tree for Rp and MAE between experimental and predicted binding affinities on antibody‐antigen dataset S630, S55 and S685. TABLE S2: Performance for PLD‐Tree of Rp and MAE on unclassified cross‐validation by different features on the considered datasets. TABLE S3: Performance of PLD‐Tree for Rp and MAE by different features on the SKEMPI v2 dataset. TABLE S4: Performance of PLD‐Tree for Rp and MAE on P2P for hyperparameter finding (1). TABLE S5: Performance of PLD‐Tree for Rp and MAE on P2P for hyperparameter finding (2). TABLE S6: Performance of PLD‐Tree for Rp and MAE on P2P for hyperparameter finding (3). FIGURE S1: Distributions of binding affinities among the three dataset PDBbind V2020, SKEMPI wt and SKEMPI mt. FIGURE S2: Scatter plots of performance using the PLD‐Tree model on the training datasets for PDBbind V2020 and SKEMPI V2. FIGURE S3: Scatter plots of performance using the PLD‐Tree model on the subset of P2P: P2P wt and P2P mt. FIGURE S4: Distributions of binding affinities among antibody–antigen test dataset S630, S55, and S685. [file PRO-34-e70377-s001.pdf]

Supplementary Material
PLNet: Persistent Laplacian Neural Network for Protein-Protein Binding Free Energy Prediction

Xingjian Xu[1], Chunmei Wang[1]*, Guo-Wei Wei[2,3,4] and Jiahui Chen[5]†

[1] Department of Mathematics, University of Florida, Gainesville, FL, 32611, USA

[2] Department of Mathematics, Michigan State University, MI 48824, USA

[3] Department of Electrical and Computer Engineering, Michigan State University, MI 48824, USA

[4] Department of Biochemistry and Molecular Biology, Michigan State University, MI 48824, USA

[5]Department of Mathematical Sciences, University of Arkansas, Fayetteville, AR 72701, USA

*Corresponding author. Email: chunmei.wang@ufl.edu

†Corresponding author. Email: jiahuic@uark.edu

Contents

1 Machine Learning model **iii**
1.1 Auxiliary features . . . iii
1.1.1 Surface areas . . . iii
1.1.2 Partial changes . . . iii
1.1.3 Atomic pairwise interaction interactions . . . iii
1.1.4 Electrostatic solvation free energy . . . iii
1.2 Training process description . . . iv
1.3 Machine Learning Models . . . iv

2 Performances on different training sets **iv**
2.1 The performance for fundamental statistical information . . . iv
2.2 The performance for cross-validation on training sets . . . v
2.3 The performance for cross-validation on S630, S55, S685 . . . v
2.4 Performance on unclassified Cross-validation . . . v
2.5 Performance on SKEMPI v2 only . . . vii
2.6 The Nested Cross-Validation Analysis . . . vii

S1 Machine Learning model

The machine learning model is presented below: the Gradient Boosting Decision Tree (GBDT), which serves as the core component of our PLD-Tree model. In the following we will introduce the computation details about auxiliary features and the training process by using GBDT.

S1.1 Auxiliary features

In the main content, we provide detailed discussions on element-specific and residue-specific persistent homology. Here, we briefly outline the generation of auxiliary features. These auxiliary features, which include other chemical and physical information not yet incorporated into element-specific persistent homology, can significantly enhance the predictive performance of our machine learning models, including the PLD-Tree. These features are concatenated with topological features and those derived from large language models for training. The auxiliary features are categorized into atom-level information. Seven groups of atom types, including C, N,O, S, H, all heavy atoms, and all atoms, are considered when generating the element-type features. Meanwhile, other three atom types,i.e., mutation site atoms, all heavy atoms, and all atoms, are used when generating the general atom-level features. In the following, we mainly discuss the techniques for getting these features.

S1.1.1 Surface areas

Atom-level solvent excluded surface are computed by ESES [1].

S1.1.2 Partial changes

Partial change of each atom is generated by pdb2pqr software [5] using the Amber force field [3] for wild type and CHARMM force field [2] for mutant. The sum of the partial charges and the sum of the absolute values of partial charges for each atomic group are collected.

S1.1.3 Atomic pairwise interaction interactions

Coulomb energy of the ith single atom is calculated as the sum of pairwise Coulomb energy with every other atom as

$$C_i = \sum_{j,j\neq i} k_e \frac{q_i q_j}{r_{ij}}, \tag{1}$$

where k_e is the Coulomb's constant, r_{ij} is the distance of ith atom to jth atom, and q_i is the charge of ith atom. The van der Waals energy of the ith atom is modeled as the sum of pairwise Lennard-Jones potentials with other atoms as

$$V_i = \sum_{j,j\neq i} \epsilon \Big[\big(\frac{r_i + r_j}{r_{ij}}\big)^{12} - 2\big(\frac{r_i + r_j}{r_{ij}}\big)^{6} \Big], \tag{2}$$

where ϵ is the depth of the potential well, and r_i is van der Waals radii.

In atomic pairwise interaction, 5 groups (C, N, O, S, and all heavy atoms) are counted both for Coulomb interaction energy and van der Waals interaction energy.

S1.1.4 Electrostatic solvation free energy

Electrostatic solvation free energy of each atom is calculated using the Poisson-Boltzmann equation via MIBPB [4] and are summed up by atom groups.

S1.2 Training process description

For the databases discussed in the main content—PDBbind V2020 and SKEMPI v2—we employed a comprehensive feature extraction strategy to ensure robust model performance. Specifically, we used a topology-based method to derive PPI features, capturing critical protein-protein interaction patterns. Additionally, we calculated the pairwise intersection of atoms and incorporated advanced biophysical techniques to extract detailed biophysics features, reflecting the molecular and structural properties of the complexes. Furthermore, embeddings from a large language model (ESM) were utilized to capture sequence-level and contextual information, which is essential for understanding complex mutational and structural dynamics.

The combination of these feature sets was then used to train and evaluate the GBDT model across these datasets. By leveraging diverse yet complementary features, we aimed to enhance the predictive power and generalizability of the model. This multi-faceted approach not only bridges the gap between sequence, structure, and interaction-level insights but also underscores the importance of integrating machine learning techniques with domain-specific knowledge to achieve superior performance in predicting binding free energies. The use of the GBDT model ensures effective utilization of these diverse features while highlighting its ability to handle complex, high-dimensional data.

S1.3 Machine Learning Models

In this work, we used GBDT primarily to compare predictive accuracy and stability against a multilayer perceptron (MLP). Within our persistent Laplacian feature–based framework (PLD-Tree), the comparable performance of GBDT combined with its robustness to overfitting made it the backbone for our extended PLNet model for predicting Kd values of PPI complexes. Though GBDT is a traditional machine learning model, it offers an important advantage over random forests by not only delivering strong predictive accuracy but also selecting the most informative topological features from the input space.

To optimize GBDT performance, we also conducted a grid search over key hyperparameters of sklearn. The optimal configuration is: `n_estimators = 25000, max_depth = 7, min_samples_split = 3, learning_rate = 0.001, subsample = 0.3, and max_features = sqrt`. Small variations around these parameters did not significantly impact predictive accuracy, indicating a stable and well-tuned model. The implemented GBDT is a function from the scikit-learn package (version 0.22.2.post1) [6]. Then for PLD-Tree training, it is realized by using random seed sequence (91011, 121314, 151617, 212223, 242526, 272829) and then taking average of predicted values to assess results stability. In the following results, we also take this random seed sequence.

S2 Performances on different training sets

In the main content, we primarily use the PDBbind V2020 and SKEMPI v2 datasets for training. In Section 3.1, the performance is described for PDBbind V2020, SKEMPI wild-type (wt), SKEMPI mutant (mt), and their combined dataset. Here we will show more different performances related to these considered datasets.

S2.1 The performance for fundamental statistical information

In Figure S1, we present bar plots illustrating the binding affinity distributions among three datasets: PDBbind V2020, wt, and mt. The binding affinities predominantly fall within the range of [-18, -4] kcal/mol, with a significant concentration between [-12, -10] kcal/mol.

- PDBbind V2020 (Left): The dataset contains a broad range of binding affinities, with the highest frequency observed between -12 and -10 kcal/mol, comprising 708 complexes. Other prominent intervals include -10 to -8 kcal/mol with 691 complexes and -14 to -12 kcal/mol with 343 complexes.

- wt(Center): This dataset shows a similar trend but with smaller sample sizes. The most frequent interval remains -12 to -10 kcal/mol with 90 complexes, followed by -10 to -8 kcal/mol with 74 complexes.
- mt(Right): The dataset exhibits a distribution pattern identical to the wild-type dataset in terms of binding affinity intervals. Notably, the intervals [-14, -12] and [-12, -10] kcal/mol also dominate with 991 and 944 complexes, respectively.

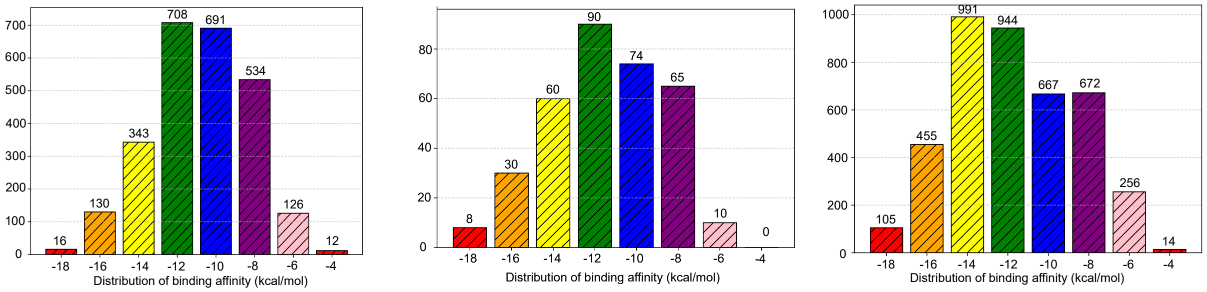

Figure S1: Distribution of binding affinity (Left: PDBbind V2020; Center: SKEMPI wt; Right: SKEMPI mt).

S2.2 The performance for cross-validation on training sets

Additionally, we perform 10-fold cross-validation on PDBbind V2020 combined with both wt and mt datasets separately. The PLD-Tree model achieved a Pearson correlation coefficient (R_p) of 0.6717 and a mean absolute error (MAE) of 1.450 kcal/mol for PDBbind V2020 when enhanced with the wild-type dataset. For P2P mt dataset, we observed an R_p of 0.7647, an MAE of 1.467 kcal/mol, and a RMSE of 1.942 kcal/mol. These results in Figure S3 demonstrate the availability of the PLD-Tree.

S2.3 The performance for cross-validation on S630, S55, S685

Table S1: The performance of PLNet and PLD-Tree for R_p and MAE between experimental and predicted binding affinities on antibody-antigen dataset.

	PLD-Tree		PLNet	
**Datasets**	R_p	MAE(kcal/mol)	R_p	MAE(kcal/mol)
S630	0.4925	1.457	0.4492	1.555
S55	0.3665	1.429	0.2922	2.014
S685	0.5259	1.395	0.4642	1.556

We report the performance of S630, S55, and S685 using a 10-fold cross-validation, as described in Section 3.4 in Figure S4 and Table S1. S630 is derived from the PDBbind V2020 dataset; S55 is extracted from the SKEMPI dataset; and S685 comes from our training set. Further details on the distributions are shown in the following figure. Although these results demonstrate potential for practical applications, we anticipate that continued improvements in feature selection and modeling techniques will lead to even greater accuracy in the future.

S2.4 Performance on unclassified Cross-validation

In this section, we present the performance results (see Table S2) for unclassified cross-validation, treating each protein, including its mutant variants, as an independent entity. In this case, the mutant dataset is directly incorporated without clustering, allowing for a unique evaluation of each protein during training. We evaluated the performance of PLD-Tree using a 10-fold cross-validation on the data sets mentioned in the main content, as shown in the following table. The notation meaning for 'V2020', 'P2P wt', 'P2P mt', 'P2P', 'Biophysics', 'PPI', 'ESM', and 'All' is the same as results in the main content. The following table demonstrates that expanding the dataset with either mutant or wild-type complexes generally improves performance, highlighting the advantages of incorporating a diverse range of protein complexes into the

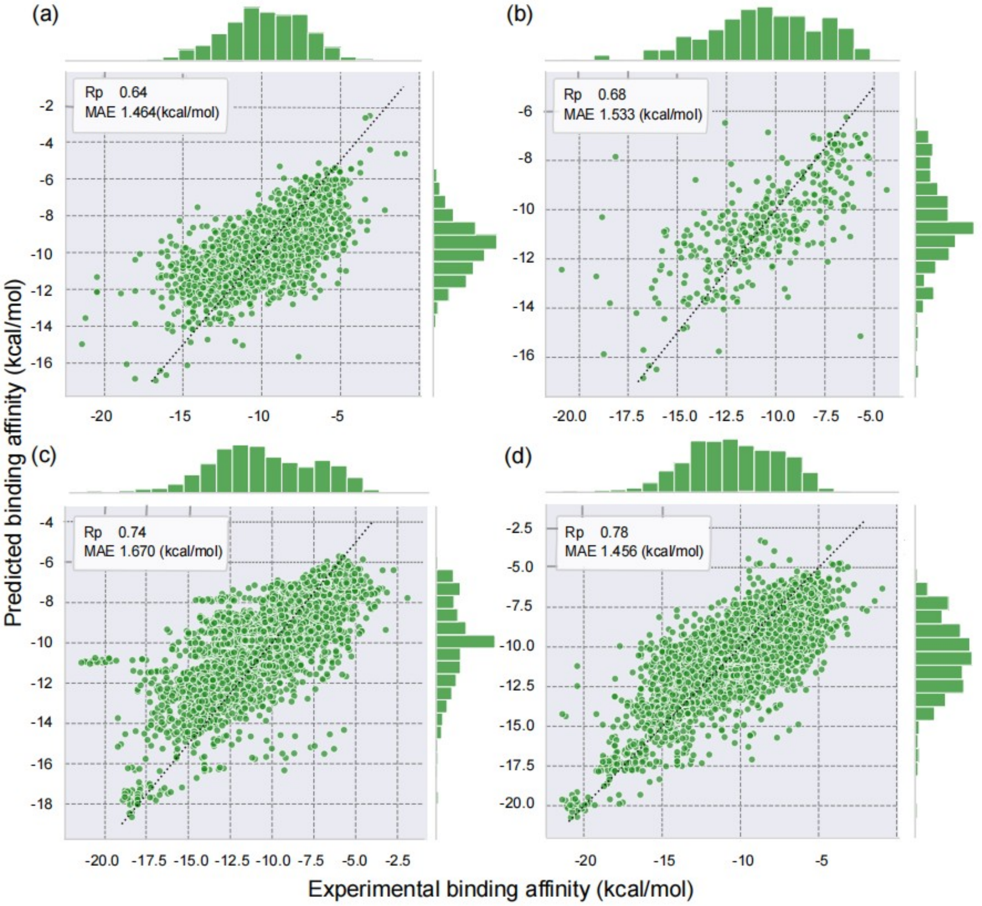

Figure S2: The performance of scatter plots using PLD-Tree model on the training sets for PDBbind V2020, and SKEMPI v2.

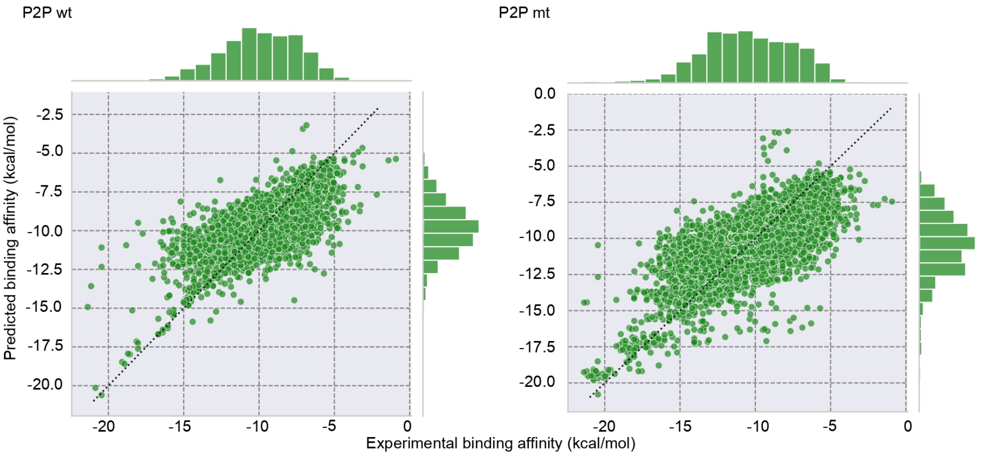

Figure S3: The performance of scatter plots using the PLD-Tree model on the training subsets of P2P. (left: P2P wt; right: P2P mt)

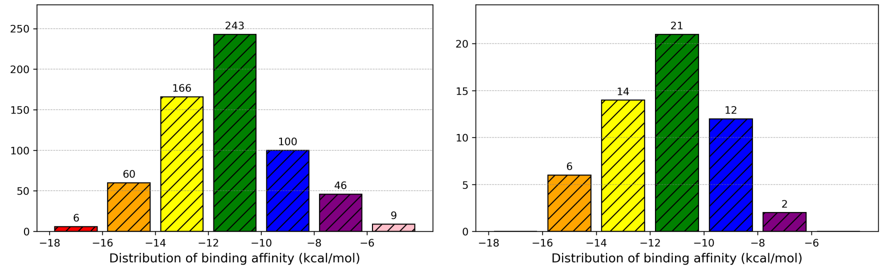

Figure S4: Distribution of binding affinity (Left: S630; Right: S55).

model.

Table S2: The performance for PLD-Tree of R_p and MAE on unclassified cross-validation by different features on the considered datasets.

	V2020		P2P wt		P2P mt		P2P	
10fold	R_p	MAE	R_p	MAE	R_p	MAE	R_p	MAE
**Auxiliary**	0.5893	1.555	0.6191	1.558	0.8521	1.106	0.8494	1.129
**PPI**	0.5617	1.610	0.5976	1.613	0.8546	1.099	0.8570	1.083
**ESM**	0.6093	1.506	0.6355	1.499	0.8598	1.088	0.8584	1.088
**All**	0.6424	1.464	0.6717	1.450	0.8755	1.018	0.8756	1.016

S2.5 Performance on SKEMPI v2 only

Although SKEMPI v2 was not analyzed in the main text, Table S3 below reports 10-fold cross-validation results for SKEMPI wt, SKEMPI mt, and SKEMPI v2 across all feature sets (Biophysics, PPI, ESM), using the same notation as in the main text. Across a range of random seeds (91011, 121314, 151617, 212223, 242526, 272829), these SKEMPI v2 configurations showed consistent gains in predictive performance. In particular, under the SKEMPI v2 setting, PLD-Tree achieved its best accuracy with R_p of 0.7810, an MAE of 1.619 kcal/mol, and RMSE of 2.119 kcal/mol in the SKEMPI v2 configuration, representing the highest accuracy in this analysis. The results highlight the importance of incorporating diverse features and datasets in improving the predictive capabilities of PLD-Tree. The combination of biophysical, interaction, and sequence-level features, along with a comprehensive dataset, significantly enhanced the model's accuracy, as reflected in the R_p and MAE values.

Table S3: Performance of PLD-Tree for R_p and MAE by different features on the SKEMPI v2 dataset.

	SKEMPI v2 wt		SKEMPI v2 mt		SKEMPI v2	
10fold	R_p	MAE	R_p	MAE	R_p	MAE
**Auxiliary**	0.6242	1.780	0.6767	1.894	0.6830	1.926
**PPI**	0.6670	1.691	0.6835	1.939	0.7077	1.880
**ESM**	0.6401	1.597	0.7199	1.714	0.7498	1.662
**All**	0.6811	1.533	0.7488	1.670	0.7810	1.619

S2.6 The Nested Cross-Validation Analysis

We also perform nested cross-validation to select hyperparameters for the GBDT model using random seed 789. To illustrate the results, we present three tables corresponding to different values of `n_estimator` (20,000 and 25,000), `depth` (from 3 to 8), and `min_samples_split` (from 2 to 5). Based on the outcomes of the nested cross-validation tests, we determine the final set of hyperparameters mentioned in the main paper that yield the best performance (see Tables S4, S5, and S6).

Table S4: The performance of PLD-Tree for the depth lists $d = 3 - 8$ with `n_estimator` 25000 and `min_samples_split` 3.

Dataset	**First row:** $d = 3, 4, 5$								
	$d = 3$			$d = 4$			$d = 5$		
	MAE	RMSE	R_p	MAE	RMSE	R_p	MAE	RMSE	R_p
P2P	1.505	1.956	0.7633	1.475	1.931	0.7710	1.463	1.923	0.7749
$P2P_{mt}$	1.510	1.971	0.7535	1.487	1.958	0.7570	1.480	1.949	0.7608
$P2P_{wt}$	1.482	1.928	0.6617	1.457	1.908	0.6695	1.449	1.904	0.6710
V2020	1.489	1.931	0.6320	1.475	1.922	0.6378	1.472	1.921	0.6378
Dataset	**Second row:** $d = 6, 7, 8$								
	$d = 6$			$d = 7$			$d = 8$		
	MAE	RMSE	R_p	MAE	RMSE	R_p	MAE	RMSE	R_p
P2P	1.461	1.926	0.7756	1.460	1.926	0.7760	1.457	1.930	0.7752
$P2P_{mt}$	1.474	1.951	0.7605	1.473	1.954	0.7590	1.478	1.965	0.7578
$P2P_{wt}$	1.447	1.902	0.6737	1.452	1.907	0.6705	1.451	1.906	0.6710
V2020	1.473	1.922	0.6370	1.472	1.921	0.6370	1.475	1.925	0.6368

Table S5: The performance of PLD-Tree for the depth lists $d = 3 - 8$ with `n_estimator` 20000 and `min_samples_split` 3.

Dataset	**First row:** $d = 3, 4, 5$								
	$d = 3$			$d = 4$			$d = 5$		
	MAE	RMSE	R_p	MAE	RMSE	R_p	MAE	RMSE	R_p
P2P	1.522	1.975	0.7593	1.490	1.945	0.7670	1.475	1.933	0.7724
$P2P_{mt}$	1.526	1.986	0.7505	1.500	1.968	0.7550	1.491	1.957	0.7586
$P2P_{wt}$	1.493	1.939	0.6577	1.466	1.915	0.6675	1.454	1.907	0.6700
V2020	1.497	1.938	0.6300	1.481	1.925	0.6368	1.474	1.921	0.6378
Dataset	**Second row:** $d = 6, 7, 8$								
	$d = 6$			$d = 7$			$d = 8$		
	MAE	RMSE	R_p	MAE	RMSE	R_p	MAE	RMSE	R_p
P2P	1.470	1.934	0.7732	1.466	1.930	0.7750	1.462	1.933	0.7747
$P2P_{mt}$	1.483	1.957	0.7593	1.479	1.958	0.7596	1.482	1.967	0.7566
$P2P_{wt}$	1.451	1.904	0.6721	1.454	1.908	0.6702	1.452	1.907	0.6710
V2020	1.475	1.922	0.6370	1.473	1.922	0.6377	1.475	1.925	0.6364

Table S6: The performance of PLD-Tree for varying `min_samples_split` (fixed `max_depth` = 7).

Dataset	split = 2			split = 3			split = 4			split = 5		
	MAE	RMSE	R_p	MAE	RMSE	R_p	MAE	RMSE	R_p	MAE	RMSE	R_p
P2P	1.463	1.933	0.7744	1.460	1.926	0.7760	1.464	1.932	0.7742	1.462	1.930	0.7754
$P2P_{mt}$	1.470	1.952	0.7602	1.473	1.954	0.7590	1.468	1.953	0.7601	1.475	1.955	0.7608
$P2P_{wt}$	1.450	1.906	0.6714	1.452	1.907	0.6705	1.448	1.904	0.6722	1.449	1.903	0.6726
V2020	1.472	1.922	0.6371	1.472	1.921	0.6370	1.470	1.921	0.6385	1.471	1.922	0.6373

References

[1] B. Liu, B. Wang, R. Zhao, Y. Tong, and G.-W. Wei. ESES: software for Eulerian solvent-excluded surface, *Journal of Computational Chemistry*, 38(7):446–466 2017.

[2] B. R. Brooks, C. L. Brooks III, A. D. Mackerell Jr, L. Nilsson, R. J. Petrella, B. Roux, Y. Won, G. Archontis, C. Bartels, S. Boresch, et al. Charmm: the biomolecular simulation program. *Journal of computational chemistry*, 30(10):1545–1614, 2009.

[3] D. A. Case, T. A. Darden, T. E. Cheatham, C. L. Simmerling, J. Wang, R. E. Duke, R. Luo, M. Crowley, R. C. Walker, W. Zhang, et al. Amber 10. Technical report, University of California, 2008.

[4] D. Chen, Z. Chen, C. Chen, W. Geng, and G.-W. Wei. MIBPB: a software package for electrostatic analysis. *Journal of computational chemistry*, 32(4):756–770, 2011.

[5] T. J. Dolinsky, J. E. Nielsen, J. A. McCammon, and N. A. Baker. Pdb2pqr: an automated pipeline for the setup of Poisson–Boltzmann electrostatic calculations. *Nucleic acids research*, 32(suppl_2):W665–W667, 2004.

[6] F. Pedregosa, G. Varoquaux, A. Gramfort, V. Michel, B. Thirion, O. Grisel, M. Blondel, P. Prettenhofer, R. Weiss, V. Dubourg, et al. Scikit-learn: Machine learning in python. *The Journal of machine Learning research*, 12:2825–2830, 2011.
